# Biosynthetic pathways and related genes regulation of bioactive ingredients in mulberry leaves

**DOI:** 10.1080/15592324.2023.2287881

**Published:** 2023-11-28

**Authors:** Na Lu, Lei Zhang, Yuqing Tian, Jinghua Yang, Shicun Zheng, Liang Wang, Wei Guo

**Affiliations:** Research Center of Traditional Chinese Medicine and Clinical Pharmacy, Shandong Provincial Maternal and Child Health Care Hospital Affiliated to Qingdao University, Jinan, China

**Keywords:** Mulberry leaves, active ingredients, secondary metabolism regulation, biosynthetic pathways, genes

## Abstract

Mulberry leaves are served not only as fodder for silkworms but also as potential functional food, exhibiting nutritional and medical benefits due to the complex and diverse constituents, including alkaloids, flavonoids, phenolic acids, and benzofurans, which possess a wide range of biological activities, such as anti-diabete, anti-oxidant, anti-inflammatory, and so on. Nevertheless, compared with the well-studied phytochemistry and pharmacology of mulberry leaves, the current understanding of the biosynthesis mechanisms and regulatory mechanisms of active ingredients in mulberry leaves remain unclear. Natural resources of these active ingredients are limited owing to their low contents in mulberry leaves tissues and the long growth cycle of mulberry. Biosynthesis is emerging as an alternative means for accumulation of the desired high-value compounds, which can broaden channels for their large-scale green productions. Therefore, this review summarizes the recent research advance on the correlative key genes, enzyme biocatalytic reactions and biosynthetic pathways of valuable natural ingredients (i.e. alkaloids, flavonoids, phenolic acids, and benzofurans) in mulberry leaves, thereby offering important insights for their further biomanufacturing.

## Introduction

*Morus alba* L., popularly known as mulberry, is a fast-growing perennial shrub belonging to the Moraceae family, which can be found in a wide range of topographical and soil conditions. Accordingly, it is widely cultivated in China, North Korea, Japan, Mongolia, Russia, India, Central Asian and European countries. The medical and nutritional benefits of mulberry have been recorded since the ancient Chinese Material Medica, for instance, mulberry fruits, the bark of mulberry stems, branches and roots, mulberry leaves and so on. Mulberry leaves are served not only as fodder for silkworms but also as potential functional food.^1–3^ In addition, mulberry leaves are one of the most common traditional Chinese medicines (TCM) aiming to dispel wind and heat, clear away lung-heat, moisten dryness, improve acuity of vision in Chinese ancient time. Modern studies revealed that mulberry leaves had exerted different active ingredients, such as alkaloids, flavonoids, phenolic acids, and benzofurans, endowing them various pharmacological properties, including anti-oxidant, anti-inflammatory, anti-aging, anti-viral, and anti-cancer, hypoglycemic activities.^4–12^

A number of reviews have described the phytochemical and pharmacological functions of mulberry.^13–17^ However, the key genes involved in metabolic pathways of multiple secondary metabolites from mulberry leaves have not been identified. In recent years, synthetic biology has become a promising strategy to produce natural bioactive compounds by utilizing the metabolic pathways of plants.^18–21^ Therefore, it is necessary to explicate the complete biosynthetic pathways and key enzyme genes of bioactive ingredients. In this review, we systematically summarize the correlative key genes, enzyme biocatalytic reactions and biosynthetic pathways of bioactive ingredients (i.e., alkaloids, flavonoids, phenolic acids, and benzofurans) in mulberry leaves to improve the understanding of the molecular mechanism of these components, which provide new insights for the further biomanufacturing.

## Biosynthesis of alkaloids

All kinds of alkaloids including pyrrolidine-type, nortropane-type and imine-type alkaloids exist in mulberry leaves. They all have 2–5 hydroxyl groups in their molecules, and some hydroxyl groups are linked with glucose and galactose to form α- or β-glycoside. Among them, imine-type alkaloids are considered as key polyhydroxylated alkaloids that possess healthy properties, which are pyranose analogues with the nitrogen atom substitutes for the oxygen atom of the ring. 1-deoxynojirimycin (DNJ), a representative of imine-type alkaloid, is the most abundant polyhydroxylated alkaloid in mulberry leaves, and others are its derivatives. DNJ is a strong α-glycosidase inhibitor, which affects the metabolism of carbohydrates in the small intestine, the secretion of glycoproteins, cell to cell or cell to virus recognition, and other biological processes by inhibiting the activity of glycosidase.^3;22^ Thus, exploiting health food and medicine with DNJ as active ingredient has broad application prospects.

**Figure 1.**
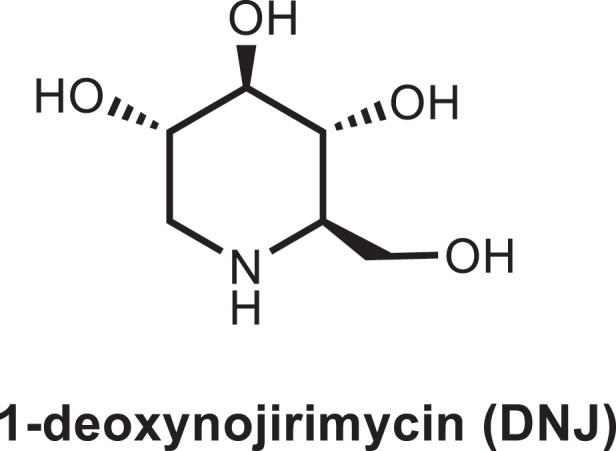
Molecular structure of DNJ in mulberry leaves.

Nishikaw et al.^23^ first isolated nojirimycin from the fermentation broth of *Streptomyces roseochromogenes R*-468 in 1965, and Inouye et al.^24^ identified its structure in 1966. Subsequently, Paulsen et al.^25^ synthesized 1,5-imino-1,5-dideoxy-D-glucit (DNJ) in 1967. Moranoline, which was the first natural DNJ, was later extracted from *Mori Cortex* in 1976.^26^ Afterward, DNJ was isolated and identified from the culture filtrate of *Streptomyces lavendulae* GC-148,^27^ the bulbs of hyacinths,^28^ and *Commelina communis* L.^29^. DNJ was also found in various tissues of mulberry trees. Until now, more than 20 polyhydroxylated alkaloids were identified in mulberry.^30–32^ Structurally, DNJ contains a scaffold of piperidine ring derived from lysine^33^ and lacks a hydroxyl group at the C1 position (Figure 1). Although mulberry trees have the highest content of DNJ among the currently discovered plants containing DNJ,^3,34,35^ the content is still too lower to meet the demand of market by traditional extraction methods, which limits the research of structure-function relationships.

**Figure 2.**
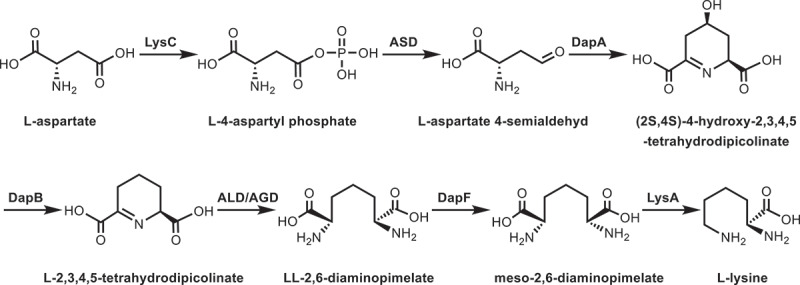
DAP pathway for lysine biosynthesis. LysC, aspartate kinase; ASD, aspartate-semialdehyde dehydrogenase; DapA, 4-hydroxy-tetrahydrodipicolinate synthase; DapB, 4-hydroxy-tetrahydrodipicolinate reductase; ALD/AGD, LL-diaminopimelate aminotransferase; DapF, diaminopimelate epimerase; LysA, diaminopimelate decarboxylase.

Identification of key genes involved in DNJ alkaloid biosynthesis and clarification of its biosynthetic pathway will ultimately realize the synthetic biological production. The Kyoto Encyclopedia of Genes and Genomes (KEGG) is a database integrating genomic, chemical and system functional information. The biggest feature is to associate the gene catalog obtained from the fully sequenced genome with the system functions at higher levels of cells, species and ecosystems. Furthermore, KEGG also has a powerful graphic function to clarify many metabolic pathways and describe the detailed information of compounds, key enzymes, and functional genes in the pathway. The diaminopimelic acid (DAP) pathway identified by KEGG begins with L-aspartate as substrate and exists in most green plants to produce lysine^36^ (Figure 2). Then, lysine, as a precursor, is used for the biosynthesis of piperidine alkaloids.

**Figure 3.**
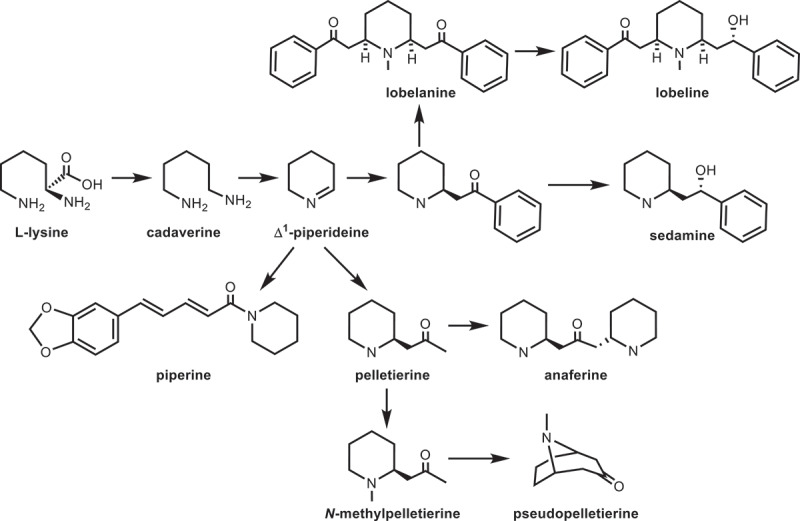
Biosynthetic pathway of piperidine alkaloids in KEGG database.

Recently, some significant progress has been achieved in the biosynthetic pathways of piperidine alkaloids such as piperine,^37^ sedamine,^38^ lobeline,^39^ anaferine, and pseudopelletierine,^40^ which can be seen from the metabolic pathway of piperidine alkaloids based on KEGG database (Figure 3). It possesses a single pathway in the piperidine ring biosynthesis. Previous studies have identified two enzymes: lysine decarboxylase (LDC)^41^ and primary-amine oxidase (AOC)^42^ in this pathway. Wang et al.^43^ identified 38 unique candidate genes involved in DNJ metabolism of mulberry leaves by transcriptomic analysis thus far, nine of which had remarkably different expression. Three transcripts associated with DNJ content were assigned to LDC and AOC genes. And five cytochrome P450 (CYP450) genes and two methyltransferase (MT) genes also related to DNJ accumulation. CYP450 enzymes play critical regulatory roles in the biosynthesis of mulberry secondary metabolites,^44^ which can catalyze the hydroxylation, alkylation, and alkenyl epoxides, hydrocarbon oxidation, and dealkylation of nitrogen, sulfur, oxygen sites, and hydroxylation and oxidation of nitrogen sites^43^. Wan et al.^45^ investigated the methylation step of piperidine ring in the biosynthetic pathway of DNJ for the first time. The interaction of MTs with piperidine and S-adenosyl-L-methionine (SAM) was verified by molecular docking. It is indicated that MaMT1 named serine hydroxymethyltransferase had C-methyltransferase activity using SAM as methyl donor and piperidine as substrate. Liu et al.^46^ screened short-chain dehydrogenase/reductase (SDR) genes related to DNJ content by a comparative transcriptome analysis of two samples from mulberry leaves. The reductase genes were successfully cloned and expressed in prokaryotic system. Functional analysis has shown that reductase could catalyze the D^1^-piperideine to piperidine. Similarly, Liu et al.^47^ successfully constructed gene overexpression system and virus-induced gene silencing system to verify the influence of LDC, AOC, and SDR genes on DNJ content. Finally, these genes were indicated to participate in the DNJ biosynthesis.

**Figure 4.**
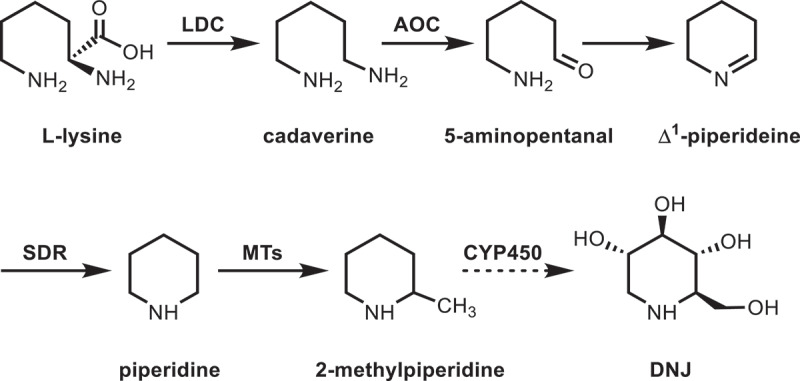
Biosynthetic pathway of DNJ alkaloid in mulberry leaves. The solid lines represent the enzymes that have been characterized clearly. The dotted lines represent possible DNJ biosynthetic enzyme.

Hence, the DNJ alkaloid biosynthetic pathway is basically elucidated with reference to piperidine alkaloids in other plants^48–53^ (Figure 4). Lysine is first catalyzed by LDC to produce cadaverine. Then oxidative deamination is occurred under the catalysis of AOC to form 5-aminopentanal. 5-aminopentanal is spontaneously cyclized to Δ^1^-piperideine. SDR is used to reduce C=N bond of Δ^1^-piperideine to generate piperidine. Subsequently, 2-methylpiperidine is formed through the C-methylation of piperidine by MTs. Presumably, DNJ might be formed through the hydroxylation of 2-methylpiperidine by CYP450. And the biosynthesis of DNJ alkaloid is ultimately achieved.

## Biosynthesis of flavonoids

**Figure 5.**
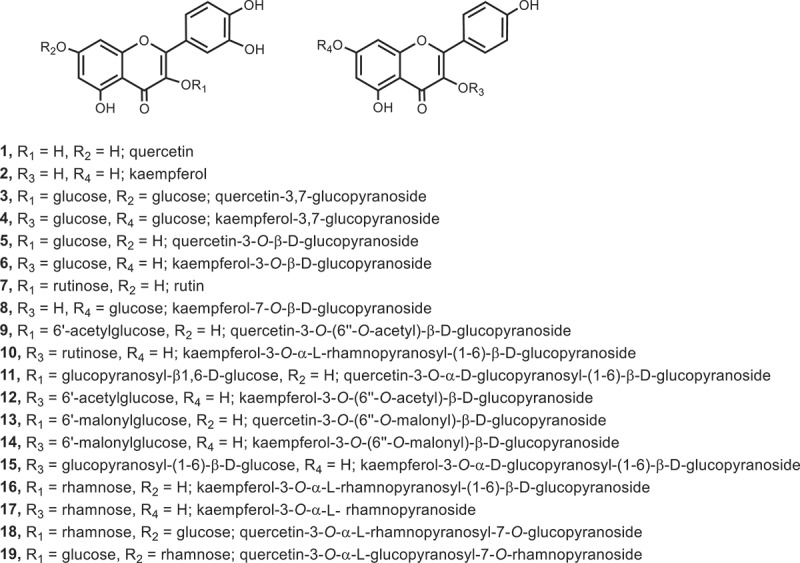
Chemical structures of flavonoids in mulberry leaves.

Flavonoids share a common diphenylpropane (C6-C3-C6) structure in which two aromatic rings are linked via a three-carbon chain.^54^ Based on the different substitution patterns of the rings, flavonoids can be further classified into several subgroups, such as flavones, flavonols, flavanones, isoflavones, and chalcones.^9,55^ In all plant stems and leaves, mulberry leaves contain the highest content of flavonoids, accounting for about 1.0%-3.0% of their dry weight.^56^ It is shown that a great number of flavonoids were isolated from the ethanol, butanol and methanol extract of mulberry leaves^7,57–62^ in previous studies, which mainly are flavonols and flavonol glycosides (i.e., rutin, quercetin, kaempferol, isoquercetin and so on, Figure 5) with a wide range of biological functions, such as lowering blood pressure, lowering blood lipid, lowering blood glucose and clearing free radicals.^5,7,63,64^

**Figure 6.**
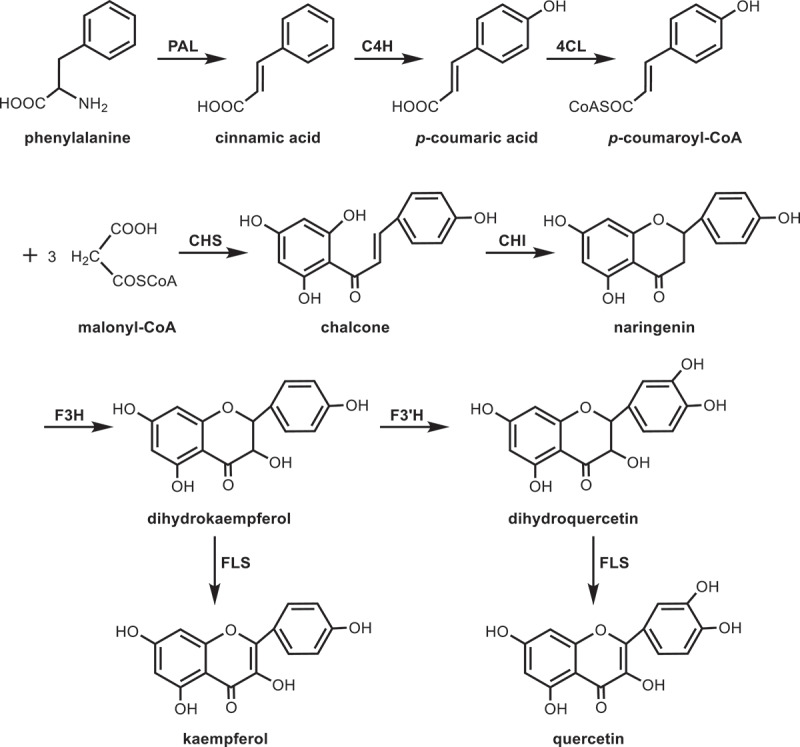
Biosynthetic pathway of flavonoids in mulberry leaves.

The biosynthetic pathway of flavonoids, as a common metabolic pathway,^65–68^ has been clearly studied in many plants. According to the draft genome sequence of *Morus notabilis*, most of the genes related to flavonoid skeleton biosynthesis have been identified.^69^ The biosynthesis of flavonoids starts from the phenylpropanoid metabolic pathway of phenylalanine, and phenylalanine ammonia-lyase (PAL) is the first key enzyme involved in the synthesis of flavonoids from mulberry leaves,^70^ which catalyzes the deamination of phenylalanine to cinnamic acid. Cinnamic acid is converted to *p*-coumaric acid by cinnamate 4-hydroxylase (C4H). 4-coumaroyl-CoA ligase (4CL) then converts coumaric acid to *p*-coumaroyl-CoA. The biosynthesis reaction between one molecule of *p*-coumaroyl-CoA and three molecules of malonyl-CoA is catalyzed by chalcone synthase (CHS). The chalcone formed is the key intermediate in flavonoid biosynthesis, which can be further derived and transformed into various flavonoids.^71^ Chalcone isomerase (CHI) can catalyze chalcone to form naringenin, which is usually the precursor of flavonol, anthocyanin, proanthocyanidin, flavone and isoflavone.^72^ Naringenin generates dihydrokaempferol under the catalysis of flavone 3-hydroxylase (F3H), and then flavonoid 3’-hydroxylase (F3’H) catalyzes the conversion of dihydrokaempferol to dihydroquercetin, and flavonol synthase (FLS) further reduces dihydrokaempferol and dihydroquercetin to kaempferol and quercetin (Figure 6).

Although the biosynthetic pathway of flavonoid skeleton in mulberry leaves has been elucidated, it is still a challenging task to identify the flavonol terminal modification genes, because its skeleton is usually terminal modified with glycosyl, malonyl, acetyl or other groups. Sugiyama et al.^73^ analyzed the distribution of quercetin 3-*O*-(6”-*O*-malonyl)-glucopyranoside and quercetin 3-*O*-(6”-*O*-acetyl)-glucopyranoside in 176 mulberry resources, and predicted that specific malonyltransferase (MaT) and acetyltransferase (ACT) were involved in the synthesis of these acylated flavonol glycosides. Zhao et al.^74^ found that *MaUGT78D1* was closely related to the accumulation of rutin in different tissues of mulberry, and speculated that flavonol-3-*O*-glucoside-L-rhamnosyltransferase (UGT78D1) played a certain part in rutin biosynthetic pathway. Li et al.^75^ confirmed that the gene encoding flavonol-3-*O*-glucoside-*O*-rhamnosyltransferase (FGRT) is a key gene in rutin formation by metabolic profiling and transcriptome analyses. *In vitro* characterization experiments showed that the enzyme was indeed involved in the synthesis of rutin, laying a foundation for the successful reconstruction of flavonoids biosynthesis in mulberry.

## Biosynthesis of phenolic acids

The total phenolic acids extracted from different species samples of mulberry leaves varied from 71.7 mg/100 g to 184.3 mg/100 g of dried plant material.^76^ Some studies have revealed that mulberry leaves contain many phenolic acids, these phenolic acids can be subdivided into two main subgroups, hydroxycinnamic acid (*m*-coumaric acid, *p*-coumaric acid, ferulic acid, sinapic acid, caffeoylquinic acids) and hydroxybenzoic acids (gallic acid, protocatechuic acid, *p*-hydroxybenzoic acid, syringic acid, vanillic acid).^76–78^ Caffeoylquinic acids are the major phenolic acids, comprising 1-caffeoylquinic acid, 3-caffeoylquinic acid (neochlorogenic acid), 4-caffeoylquinic acid (cryptochlorogenic acid), and 5-caffeoylquinic acid (chlorogenic acid, CGA). Among them, CGA is the dominant component and its abundance account for 60.5%-67.2% of the total phenolic acids.^76^

**Figure 7.**
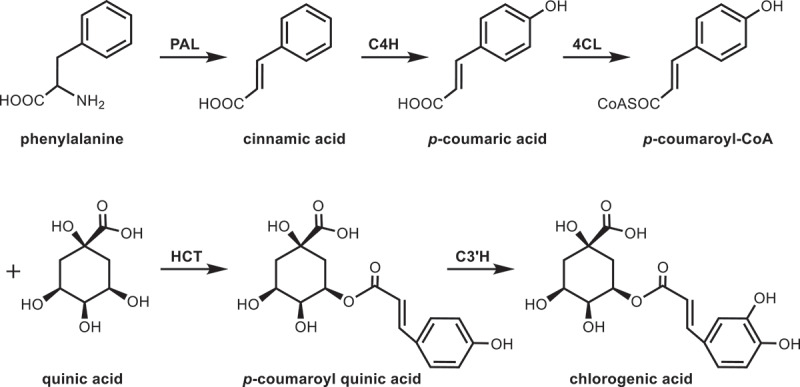
Biosynthetic pathway of chlorogenic acid in mulberry leaves.

The biosynthesis of CGA origin from the aromatic amino acid phenylalanine. The first three steps of enzyme catalysis for the conversion of phenylalanine to CGA are the same as the synthesis of aromatic secondary metabolites, including stereospecific deamination of phenylalanine, hydroxylation of cinnamic acid at position 4 and 4-coumaroyl-CoA ligase reaction.^79^ Zhao et al.^80^ performed transcriptome sequencing on two mulberry leaf samples with different CGA contents (before and after frost) and found that 58 genes encoded five enzymes were annotated in the CGA biosynthetic pathway. Among them, 17 genes related to CGA biosynthesis were differentially expressed. Correlation analysis revealed that the expression levels of only four genes were significantly positively correlated with CGA accumulation, including those encoding PAL, 4CL, hydroxycinnamoyl-CoA shikimate/quinate hydroxycinnamoyltransferase (HCT), and coumaroyl quinate/shikimate 3’-hydroxylase (C3’H), indicating that they are key genes in CGA biosynthesis of mulberry leaves. Meanwhile, they cloned and characterized *MaHCT4* from mulberry leaves. *In vitro* enzymatic assays confirmed that MaHCT4 mainly controlled the synthesis of *p*-coumaroyl quinic acid in mulberry leaves using *p*-coumaroyl-CoA as an acyl donor and quinic acid as acyl acceptor^80^. The possible pathway for CGA biosynthesis was clarified (Figure 7). In phenylpropanoid metabolic pathway, phenylalanine is converted by PAL, C4H, and 4CL to *p*-coumaroyl-CoA. The biosynthesis reaction between *p*-coumaroyl-CoA and quinic acid is catalyzed by HCT. Then C3’H catalyzes *p*-coumaroyl quinic acid to form chlorogenic acid.

## Biosynthesis of benzofurans

**Figure 8.**
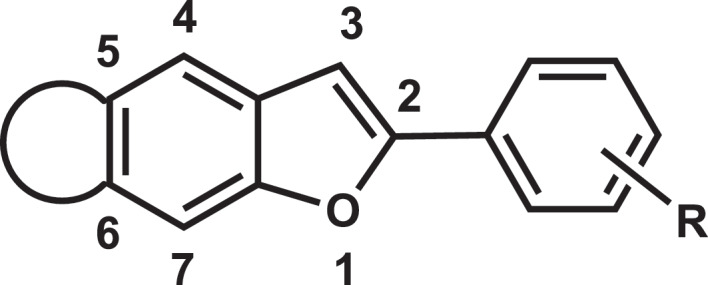
Basic backbone of moracins.

Benzofuran derivatives are the key bioactive components found in mulberry leaves,^60,62,81,82^ which exhibit wide biological properties including analgesic, antibacterial, antiviral and anti-inflammatory. The majority of them are moracins and their derivatives.^77^ Structurally, moracins contain a 2-phenyl substituted benzofuran-fused-ring (Figure 8). 26 different kinds of moracins (A-Z) have been isolated from mulberry at present, and some chemical methods have been developed to synthesize moracins.^83^ However, the challenges in isolation and synthesis still existed and restricted the development of medicinal potential. Therefore, there is an urgent need to clarify the biosynthetic pathway and regulatory mechanism of moracins.

**Figure 9.**
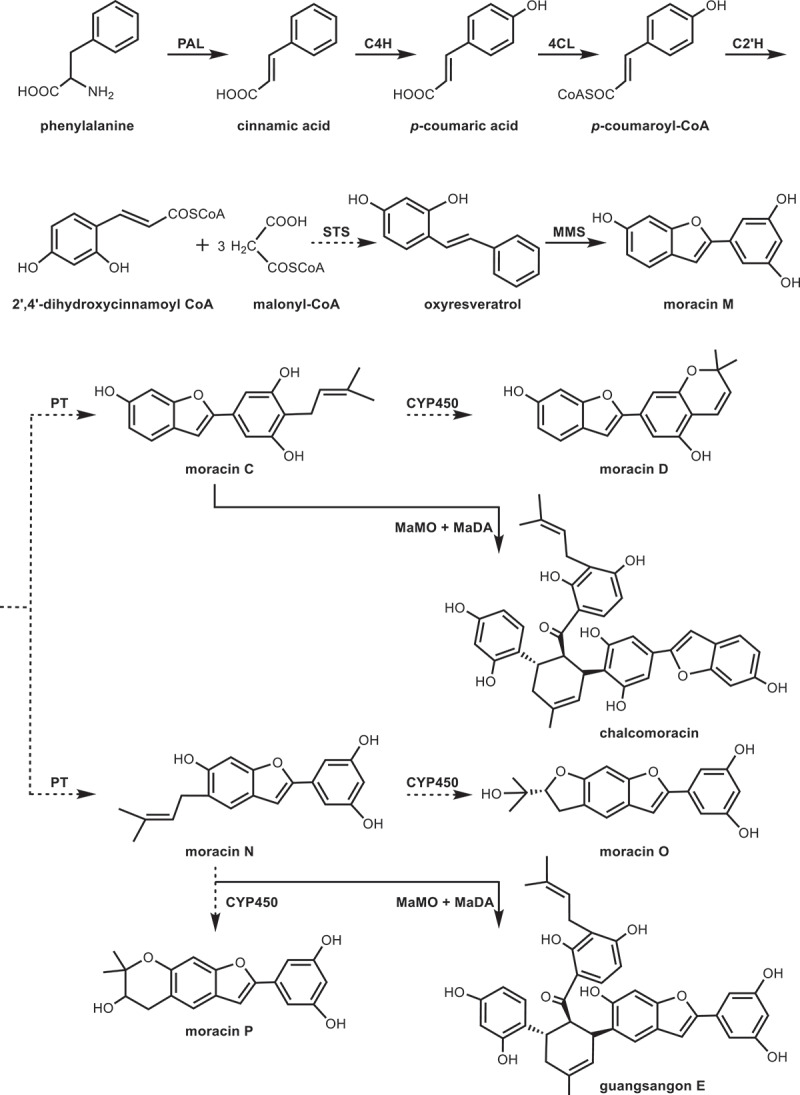
Biosynthetic pathway of moracins in mulberry leaves. The solid lines represent the enzymes that have been characterized clearly. The dotted lines represent putative biosynthetic enzyme.

Liu et al.^84^ performed *de novo* transcriptome sequencing toward different tissues of mulberry leaves. Candidate genes involved in moracins biosynthesis were identified by differential expression analysis and coexpression analysis. And a putative biosynthetic pathway was proposed (Figure 9). They speculated that moracins derived from phenylpropanoid pathway. Phenylalanine is converted to *p*-coumaroyl-CoA under the catalyzation of three enzymes PAL, C4H, and 4CL. Subsequently, *p*-coumaroyl CoA 2’-hydroxylase (C2’H) catalyzes *p*-coumaroyl-CoA to form 2’,4’-dihydroxycinnamoyl CoA. The biosynthesis reaction between one molecule of 2’,4’-dihydroxycinnamoyl CoA and three molecules of malonyl-CoA is catalyzed by stilbene synthase (STS) to form oxyresveratrol. Oxyresveratrol is currently the only identified stilbene from mulberry leaves. It shares a similar structure with moracin M, and can synthesize moracin M by moracin M synthase (MMS). Moracin M possesses the simplest structure and is considered as the key precursor for generating other moracins. Prenylation and oxidative cyclization are the most common modifications during the moracins biosynthesis. According to the Pfam annotation, liu et al.^84^ identified prenyltransferases (PTs) which could catalyze the prenylation of moracins and verified cytochrome P450s (CYP450s) responsible for the formation of furan and pyran rings. Moracin M convered to moracin C or moracin N in the presence of PTs. CYP450 catalyzed moracin C or moracin N to produce moracin D or moracin O. Besides that, two unigenes coding *Morus alba* moracin C oxidase (MaMO) and *Morus alba* Diels-Alderase (MaDA) were also identified in the downstream pathway of moracins. Moracin-derived Diels-Alder adducts, such as guangsangon E and chalcomoracin, was synthesized. Moreover, the current biosynthesis research of benzofurans is still in its infancy, further investigation on the functional characterization and transcriptional regulation of mulberry moracins biosynthesis is demanded in the future.

## Conclusions and prospects

Mulberry leaves comprise a variety of natural components with remarkable pharmacological activity, such components, featuring diversified and complex structures, can be used for research and development of innovative drugs. At present, it is complex, expensive and environmentally unfriendly to extract directly from mulberry leaves or to synthesize by traditional chemical methods. With the development of synthetic biology technology, the reconstruction of plant metabolic pathways in heterologous host organisms has become a hotspot. The advances of the genomic and transcriptomic technology, bioinformatic method and metabolomics analysis have greatly promoted the elucidation of key enzyme genes related to plant biosynthetic pathway. This review has highlighted the correlated genes and enzyme biocatalytic reactions responsible for the biosynthetic processes and proposed possible biosynthetic pathways of components (i.e., alkaloids, flavonoids, phenolic acids, and benzofurans) in mulberry leaves, which laid a foundation for realizing the production of phytochemicals by synthetic biology and greatly promoted the further development of the medicinal and nutritional value of mulberry leaves. Collectively, the review provides technical support for the development of new functional foods and drugs with effective ingredients as raw materials.

It is still hard to achieve the biomanufacturing of natural products in TCM through heterologous synthesis. Mainly because the entire biosynthetic pathways have not been well-understood and key enzyme functions and mechanisms have not been fully revealed. Accordingly, it is necessary to establish an appropriate experimental system, such as plant cell suspension culture, *Agrobacterium tumefaciens* induced root culture, specialized organs (secretory glandular hairs). The accumulation of bioactive components is easily influenced by environmental, biological and abiotic factors, and the level of metabolites is usually closely related to the expression of catalytic elements in the biosynthetic pathway. Therefore, differential analysis based on the combination of comparative transcriptome and metabolome, as well as coexpression network analysis, provide effective means for screening candidate gene elements. Genomics and proteomics techniques are also used to explore enzymes involved in specific secondary metabolic pathways, clone corresponding coding genes, perform gene function verification and gene expression regulation research, clarify specific secondary metabolic pathways and their regulatory mechanisms, and on this basis, carry out molecular regulation of metabolic pathways to increase the content of target secondary metabolites. The combination of multiple omics techniques will be widely applied in the screening of gene elements involved in the biosynthetic pathways of bioactive ingredients in TCM. Furthermore, the selection and modification of chassis cells are also an important link in the heterologous biosynthesis. At present, heterologous plants, *Escherichia coli*, *Bacillus subtilis*, *Pichia pastoris* and *Saccharomyces cerevisiae*, are the main chassis cells in synthetic biology. They possess quite complex systems, and the introduction of exogenous biological elements and pathways will be influenced by the existing metabolic and regulatory pathways in the chassis system, which cause the instability of plasmid, cell lysis and changes in the cellular genetic information, thereby affecting cell growth and target compounds production. Some factors should be considered, such as the host metabolism and the feasibility of using genetic technology in the heterologous host, the known of genomic sequences, the safety of host cells and similarity between gene expression and cellular environment. Despite the increasing depth and width of multiple omics and bioinformatics technology, there are still a large number of candidate genes waiting for functional verification, new methods are urgently needed to improve the screening rate and accuracy of candidate genes. High throughput screening, molecular probe technology, cell-free systems, computer simulation and artificial intelligence technology provide research templates and novel research ideas for the study of functional genes and have gradually been applied to the biosynthesis of active ingredients in TCM, which greatly promote the elucidation of natural products biosynthetic pathways. The elucidation of natural products biosynthetic pathways will provide rich catalytic elements for the research of natural products synthesis biology, and provide new compounds sources for the production and research of natural drugs. At the same time, pathway analysis will also provide “functional molecular markers” for the molecular breeding research of Chinese herbal medicines, accelerate the selection of excellent varieties, and promote the development of traditional Chinese medicine agriculture.
